# Review article: Evaluating the effectiveness of arterial pressure point techniques as a first aid method for external haemorrhage control: A systematic review

**DOI:** 10.1111/1742-6723.14537

**Published:** 2024-12-03

**Authors:** Zoe Jane Rodgers, Karolina Bejmert, Tiani Chung, James Furness, Philip Abery, Kevin Kemp‐Smith, Nicholas Taylor, Kimberley Casey Bruce, Peter James Snelling

**Affiliations:** ^1^ Physiotherapy Department Bond University Gold Coast Queensland Australia; ^2^ Emergency Department Canberra Hospital Canberra Australian Capital Territory Australia; ^3^ Emergency Department Gold Coast University Hospital Southport Queensland Australia; ^4^ School of Medicine and Dentistry Griffith University Southport Queensland Australia

**Keywords:** anatomical junction, arterial bleeding, limb, manual pressure, tourniquet

## Abstract

The current ANZCOR guidelines for first aid management of life‐threatening bleeding from a limb, where bleeding cannot be controlled with direct pressure, recommends the use of an arterial tourniquet. However, tourniquets required specialised training and equipment, which may not be accessible in all emergencies. This systematic review evaluated the effectiveness of arterial pressure point techniques (APPT) as a first aid measure for controlling life‐threatening, non‐compressible bleeding from limbs and anatomical junctions. A comprehensive literature search was conducted following the PRISMA guidelines. The search was used in five databases: PubMed, CINAHL, SportDiscuss, Proquest Central and Embase. Eligible studies included adult participants in and out of hospital settings, focusing on extremities and junctional areas. Studies assessing APPT alone or compared with other first aid techniques were included. The review included nine quasi‐experimental articles, with eight having low levels of evidence. Although most reported high success rates (87.5–100%) for APPT achieving blood flow cessation, its effectiveness compared to alternative methods, such as arterial tourniquets, remains inconclusive because of methodological heterogeneity and differing success benchmarks. APPT shows promise in external haemorrhage control. Additional research with higher levels of evidence, standardised protocols and larger sample sizes is needed. Investigation in real‐world scenarios is crucial to compare methods like tourniquets. Future research will determine APPT's effectiveness and its potential role as a bridging technique before tourniquet application or medical assistance.

## Graphical abstract

Despite high success rates in blood flow cessation, arterial pressure point techniques (APPT) present inconclusive efficacy compared to arterial tourniquets. Methodological heterogeneity hinders conclusive assessments, urging the need for further research with standardised protocols and higher‐level evidence to determine APPT's role in emergency haemorrhage management.

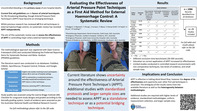

**Key findings**

- The current ANZCOR guidelines for first aid management of life‐threatening bleeding from a limb is the application of an arterial tourniquet but these can be inaccessible and require training.
- Arterial pressure point techniques (APPTs) have shown promise as an alternative to tourniquets in this context, but current evidence is limited by heterogeneity in methodology and outcome measures.
- Further research is warranted, including well designed randomised controlled trials and standardised reporting, to determine APPTs effectiveness in comparison to tourniquets as a first aid measure for haemorrhage control.

## Introduction

Trauma is the primary cause of injury‐related morbidity and mortality globally, with uncontrolled bleeding responsible for approximately 35% of trauma‐related deaths, often occurring before medical intervention.^1^, ^2^ External haemorrhages from traumatic injuries are highly preventable.^3^, ^4^ Experimental studies have suggested that over 60% of blood volume can be lost within a minute of uncontrolled arterial bleeding,^5^ underscoring the critical need for effective first aid haemorrhage control. Although existing guidelines advocate direct pressure as a first aid measure for external haemorrhage, this is often ineffective where there is life‐threatening bleeding from a limb. Arterial tourniquets are currently recommended in this situation as the first step, but it has been recognised that significant blood loss can occur before and during its use. Additionally, commercial tourniquets can be poorly available and require training to apply. ^5^, ^6^ Hence, arterial pressure point techniques have been proposed as a potential alternative for controlling external haemorrhage.^5^

Arterial pressure point techniques halt blood flow by compressing specific anatomical locations where arteries can be pressed against bony structures,^6^ offering the advantage of accessibility without specialised training or equipment.^1^ However, current first aid guidelines predominantly endorse tourniquet application because of documented concerns about caregiver fatigue and collateral circulation leading to distal pulse return.^5^, ^7^

Charlton *et al*.^1^ conducted a 2019 systematic review (SR) comparing first aid techniques for life‐threatening bleeding, favouring commercial tourniquets over manual pressure techniques or haemostatic dressings.^1^ However, no subsequent review has specifically addressed pressure point techniques. Newer articles have emerged since Charlton *et al*.'s review, further highlighting the conflicting evidence regarding manual pressure point techniques.^3^, ^5^, ^8^, ^9^, ^10^ Although some studies support the efficacy of manual compression,^1^, ^3^, ^5^, ^6^, ^8^ others note its variability influenced by factors including anatomical location and application technique.^7^, ^11^

Swan *et al*.^7^ noted the absence of studies on the efficacy, feasibility and safety of pressure point techniques in controlling bleeding. Pain to caregivers and patients emerged as a significant limitation, potentially hindering effectiveness, especially during prolonged application.^3^ A proof‐of‐concept study explored the ‘martial arts knee mount position’ as a potential strategy to alleviate caregiver fatigue before tourniquet application or while awaiting first aid personnel.^5^ However, as the study was conducted in a controlled environment, its practical effectiveness in real‐life situations remains uncertain.^5^ Another study involving 38 caregivers found that manual pressure point techniques effectively halted distal pulses, but its efficacy varied based on provider training.^3^

Recent studies warrant an updated review of evidence on pressure point techniques for traumatic arterial bleeding cessation. The objective of this SR was to evaluate the effectiveness of arterial pressure point techniques as a first aid measure in controlling life‐threatening bleeding from a limb, anatomical junctions and non‐compressible sites.

## Methods

To standardise terminology, this SR introduced the term ‘arterial pressure point techniques’ (APPT). External haemorrhage refers to bleeding from a limb or an anatomical junction (where a limb meets the trunk) at non‐compressible sites. The SR adhered to the Preferred Reporting Items for Systematic Reviews and Meta‐Analyses (PRISMA) guidelines and protocol, and the registration was completed through the Open Science Framework (OSF) on September 25th, 2023 (https://doi.org/10.17605/OSF.IO/FH3VK).

### Literature search

In September 2023, a preliminary search was conducted using PubMed to review all available research on ‘pressure points’, ‘manual compression’ and ‘external haemorrhage’. This provided insights into established literature and key terms, which were then integrated with appropriate synonyms and relevant Medical Subject Heading (MeSH) terms to create the final search strategy in collaboration with a librarian from Bond University (Supporting Information ‐ Appendix S1).

A search polyglot tool from SR accelerator^12^ was used to adapt the PubMed search strategy for use in other databases, including CINAHL, SportDiscuss, Proquest Central and Embase (Supporting Information ‐ Appendix S2). MeSH terms were modified appropriately for these databases. Clinical trial registries were also screened (Australian New Zealand Clinical Trials Registry ANZCTR and clinicaltrials.gov). Google Scholar was used to search for grey literature with guidance from CADTH Grey Matters^13^ through the following search strategy: ‘direct manual pressure point technique for external haemorrhages and first aid’. The first 100 results were screened.

### Study selection

All articles obtained from the search strategy were exported into Microsoft Endnote (20.6 Clarivate) by one reviewer (K.B.). SR Accelerator's ‘Deduplicator’ tool^14^ was used to remove duplicates. After de‐duplication, all records were exported into Endnote as per the eligibility criteria (refer to Table 1). Two reviewers (K.B. and Z.R.) independently screened the titles and abstracts of all records. From these records, the reports met the inclusion criteria and underwent a detailed full‐text screening. Final inclusion into the SR was depicted based on the explicit eligibility criteria. Excluded records were categorised appropriately with a documented rationale. Any reviewer disagreements were resolved by a third reviewer (T.C.), with the assistance of the ‘Disputatron’ tool from SR Accelerator.^14^ The study selection process ensured the reduction of selector, inclusion and recorder error bias. A PRISMA Flow Diagram^15^ was utilised to ensure proper documentation of study selection and complete transparency.

**TABLE 1:** Eligibility criteria used for article selection used to include or exclude studies

Inclusion		Exclusion
Adults 18 years and older Human participants Trained and non‐trained professionals Upper and lower limbs Junctional areas APPT compared to any other intervention or APPT alone	Publications of any year English text Quantitative studies Studies available in full‐text Peer‐reviewed studies Clinical and non‐clinical settings	Children/paediatric/adolescent/under the age of 18 Animal studies Non‐English texts Head, neck, truncal, genital and aortic areas Systematic reviews, protocols Qualitative studies, editorials

### Eligibility criteria

The PICOS framework developed by the PRISMA guidelines^15^ was used to generate the eligibility criteria (Table 1).

*Population*: Healthy adults with simulated or non‐simulated external haemorrhage both in‐ and out‐of‐hospital settings. Any study focused on the extremities or junctional areas were included, whereas nasal, oral, aortic or truncal areas were excluded.
*Intervention*: APPT applied to major upper and lower limb arteries or junctional areas.
*Comparison*: APPT alone or any other external haemorrhage management technique if a comparison was available.
*Outcomes*: The primary outcome was the reduction or cessation of blood flow, which included any of the following surrogate measures: blood velocity, blood pressure or distal pulse palpation. Secondary outcomes included training provided for technique, duration of technique, responder fatigue, adverse effects (e.g. bruising), satisfaction rating, ease of application of technique, participant reported pain or tolerability, type of clothing worn by the participant, mortality rates and tissue or organ death, physiologic response to blood transfusion.
*Study designs*: All primary quantitative study designs were incorporated (observational and experimental), including trial protocols and grey literature. Abstracts were excluded, with only full‐text articles incorporated. Only studies in English were utilised; all other languages were excluded. Secondary study designs, such as clinical guidelines and other SRs were excluded.

### Critical appraisal

The Joanna Briggs Institute (JBI) critical appraisal tool^16^ assessed the methodological quality for congruent study designs. Three authors (Z.R., T.C. and K.B.) convened to reach a consensus on the tool criteria applied for each study design. Question 6 was omitted from the JBI checklist for quasi experimental designs as follow up did not apply because the articles included were non‐randomised control trials or pre‐post intervention studies. Two reviewers (Z.R. and K.B.) individually appraised each article. All disagreements were resolved through a third reviewer (T.C.) who compared the checklist scores. The percentage of quality was calculated based on methodological quality proposed by Kennelly^17^.

### Data extraction

Relevant data from the final eligible studies were extracted and collated in a pre‐piloted data extraction table. The information retrieved included the title, article authors, research design, level of evidence, the study population and characteristics, methodology and protocol, the comparison intervention (if applicable), the anatomical location where APPT was applied, medical knowledge or training of the respondent and the key results identified from the outcome measures of interest. The level of evidence of each study was determined through the National Health and Medical Research Council (NHMRC) levels of evidence.^18^ Any information that was not provided within the respective articles was identified as ‘Unknown’ or ‘Not Provided’ in Table 2.

**TABLE 2:** Study characteristics of the included articles

Author (year)	Study design	Level of evidence^18^	Population	*N*	Quality rating (%)	Medical knowledge	Population characteristics (age, sex, height, weight, occupation)
Furness *et al*. (2023)^9^	Pilot randomised control trial	II	Surf lifesaver adults	8	88%	Basic first aid	M4/F4 Mean age (SD): 35.6 (10.6)†
Larraga‐Garcia *et al*. (2021)^17^	Pilot non‐randomised control trial	III‐2	Medical	54	50%	Medical practitioners	Final year medical students: *n* = 32 Doctors: *n* = 22†
Thompson *et al*. (2022)^3^	Non‐randomised experimental trial	III‐2	Healthcare professionals	38	50%	Advanced medical knowledge	M36/F2 Medics (*n* = 20) Physicians (*n* = 11) Medical students (*n* = 4) Nurses (*n* = 1) Special forces operator (*n* = 1)†
Kragh *et al*. (2013)^19^	Non‐randomised experimental trial†	III‐2	Unknown†	Unknown Interactions total: 120	50%	Not provided†	Not provided†
Pikman Gavriely *et al*. (2022)^10^	Prospective non‐randomised trial	III‐2	Male special forces medics	35	88%	No formal APPT training†	M35/F0 Mean age ± SE: 21.1 ± 1.3 Height (cm): 176 ± 5.6 Weight (kg): 75.1 ± 7.1†
Slevin *et al*. (2009)^5^	Cross‐sectional study	IV	Healthy volunteers	11	88%	Not provided†	M9/F2 Mean age (SD): 22.5 (6.3)†
Taylor and Lamond (2021)^11^	Non‐randomised experimental trial	III‐2	Healthcare background	32	100%	Varied (basic to advanced medical knowledge)	M18/F16†
Swan *et al*. (2009)^7^	Cross‐sectional study	IV	Healthy adult volunteers of either sex	10	75%	Not provided†	Mean age ± SE: 36.5 ± 6.0 Height (in): 68.1 ± 1.4 Weight (lbs): 154 ± 12†
Avital *et al*. (2023)^6^	Case report	IV	21‐year‐old healthy cadet special forces	1	77%	Responders: EMTS Models: Special force	Not provided†

† Missing information.

F, female; M, male.

### Data synthesis and analysis

As this SR aimed to critique and synthesise all available information using multiple study designs, the extrapolated data from each article varied. The various outcome measures included pre‐ and post‐intervention blood flow or blood pressure comparisons, time to distal pulse cessation, blood loss volume, ease of application and Doppler ultrasound records of reduced arterial peak systolic velocity (PSV) and mean arterial blood flow velocity (MAV).

Similar outcome measures were identified and grouped accordingly. The comparable outcome measures and homogenous data were illustrated in graphical format. Quantitative data with similar outcome measures were transformed into a uniform outcome measure where appropriate, and these results were tabulated against the quality and limitations of the study. Narrative summaries accompanied all tabulated data and were used to describe grouped outcome measures. Frequency statistics were used for the critical appraisal results.

Within the included articles there were various ways that each study measured blood flow cessation or reduction including pressure, time, volume of blood loss and distal blood flow cessation as seen in Table 3. To compare these results, the data was converted to a percentage of successful occlusion events. If the percentage of successful occlusion was not given, it was calculated by dividing the number of participants or interventions by the number of complete occlusions. For example, Swann *et al*.^7^ had 10 participants in four areas of APPT, but only seven studies achieved the reported benchmark for occlusion, making the occlusion percentage 17.5%.

**TABLE 3:** Table of methods and results of each study included

Author (year)	Intervention	Vessel occluded	Occlusion benchmark	Education/information	Measurement tool	Primary outcome(s)	Key results	
Furness *et al*. (2023)^9^	I1: AT I2: APPT	Femoral artery	NS	Infographic	Pulse wave Doppler US – PSV	Mean % reduction of blood flow	I1: 50.8% I2: 89.7%	
						Full blood flow cessation	I1: 4 (50%) I2: 7 (87.5%)	
Larraga‐Garcia *et al*. (2021)^17^	I1: APPT in 4 scenarios	(1) Femoral vein (2) External saphenous vein (3) Internal femoral artery (4) External femoral artery	Pressure to stop bleeding (mmHg): (1) 115 (2) 35 (3) 200 (4) 120	45 min seminar 2 days prior + 15 min explanation day of	Force sensors	Haemorrhage control	I1: all participants successful (100%)	
						Median usability	I1: between Somewhat agree and strongly agree	
Thompson *et al*. (2022)^3^	I1: Supraclavicular Manual pressure point I2: Femoral Manual pressure point	Subclavian and femoral arteries	1 min	2 min instructional presentation	Manual radial and popliteal pulse palpation	Success to DBFC	I1: 38/38 I2: 38/38	
						Median time to DBFC	I1: 3.0 s I2: 4.5 s	
						Pain (0–10, 10 severe)	I1: 3 I2: 2	
Kragh *et al*. (2013)^19^	I1: Digital compression I2: Manual compression (heel of the hand) I3: Knee compression I4: Compression with a 50 lb. kettlebell I5: CRoC I6: SAM I7: JETT I8: AAT	Common femoral artery	Time: 300 s Blood loss: 5 L	N/A	Visual cessation of flow from vessel lumen	Haemorrhage control efficacy	I1‐8: 100%	
						Average time to stop bleeding (s)	l1: 9 l2: 27 l3: 40 I4: 29	I5: 59 I6: 26 I7: 41 I8: 102
						Average blood loss volume (mL)	I1: 96 I2: 239 I3: 251 I4: 140	I5: 581 I6: 35 I7: 342 I8: 787

DBFC, distal blood flow cessation; I1–8, intervention # 1–8; N/A, not applicable; NS, not specified; PSV, peak systolic velocity; US, ultrasound; VAS, visual analog scale.

## Results

**Figure 1:**
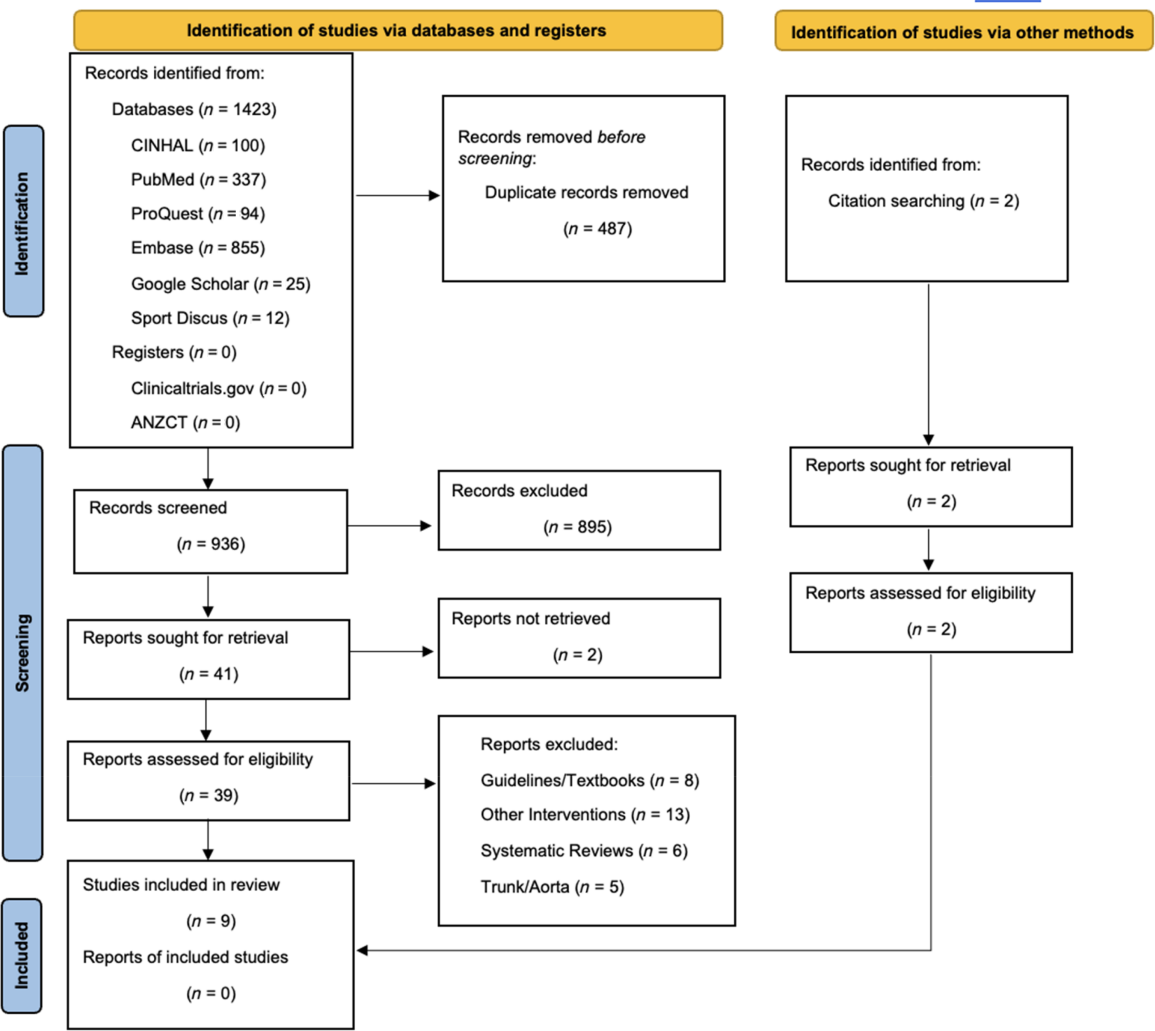
PRISMA flow diagram.

The PRISMA Flow Diagram^15^ is shown in Figure 1. A total of 1423 records were sourced from all databases.

### Study selection

After full‐text screening, nine studies met the eligibility criteria. Based on the exclusion criteria, animal studies and studies with participants under 18 were not included as they did not meet the population of interest. Qualitative and systematic reviews were excluded as primary sources were the area of interest. Lastly, studies including areas of compression at the head, neck, trunk, genitals and aortic systems were excluded because of the complexity of these systems and are less frequently seen in first‐aid situations. The PRISMA Flow Diagram^15^ (Fig. 1) illustrates the literature identification and screening process. Inter‐rater reliability, assessed using the SR Accelerator's ‘Disputatron’ tool, showed 67% agreement, which indicated a ‘fair’ agreement.

### Study characteristics and key findings

The data extracted from the studies included are provided in Tables 2, 3. There were seven quasi‐experimental studies,^3^, ^5^, ^7^, ^10^, ^11^, ^17^, ^19^ one RCT,^9^ and one case report.^6^ One study^10^ only included male participants, whereas four studies^3^, ^5^, ^9^, ^11^ included male and female participants according to biological sex. Four studies^6^, ^7^, ^17^, ^19^ did not specify the sex or gender of the participants. Five studies^3^, ^6^, ^9^, ^11^, ^17^ had a population with various medical knowledge ranging from basic first aid to medical professionals. The participant sample size of the studies varied from 1 to 38. One study^19^ did not specify the participant sample size and instead had the number of interactions for each intervention.

Five studies^3^, ^5^, ^6^, ^10^, ^17^ had interventions that included APPT in one or more locations, whereas four studies^7^, ^9^, ^11^, ^19^ included comparators, such as arterial tourniquets. Eight^3^, ^5^, ^7^, ^9^, ^10^, ^11^, ^17^, ^19^ studies assessed the femoral artery, six^3^, ^5^, ^7^, ^10^, ^11^, ^17^ of these also assessed other vessels. Only the case report^6^ did not include the femoral artery. Six of the included studies^3^, ^5^, ^7^, ^10^, ^17^, ^19^ had differing benchmarks for vessel occlusion, which varied from time to distal pulse cessation, pressure applied and blood loss. Education comprised of short (2–15 min) instructional presentations prior to application,^3^, ^10^, ^11^ infographics,^9^ and a 45 min seminar 2 days prior.^17^

### Effectiveness of APPT for blood flow reduction or cessation

**Figure 2:**
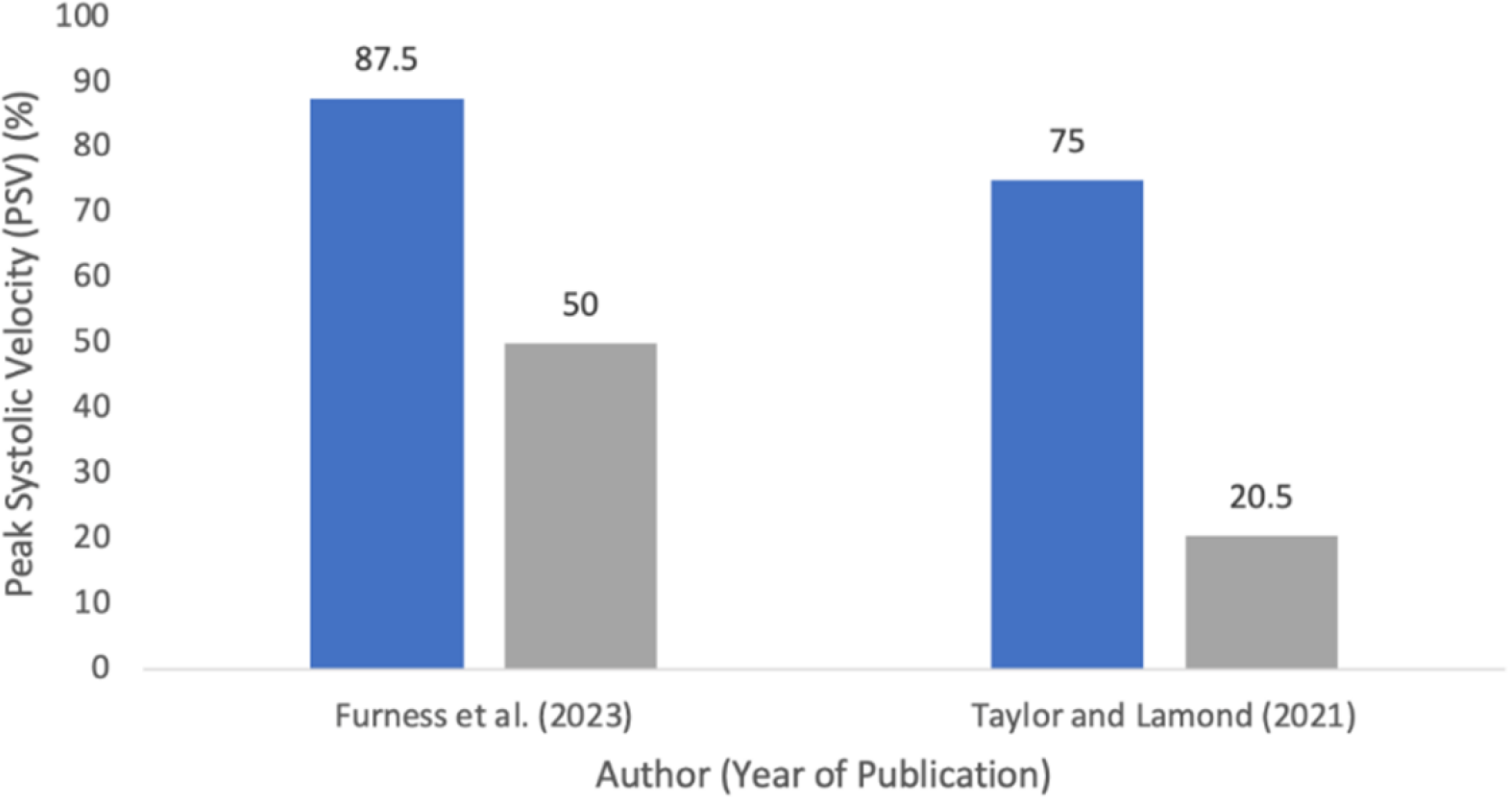
Complete reduction of blood flow as recorded by peak systolic velocity (PSV). Peak systolic velocity (PSV) is represented as percentages (%) for APPT or Leg Rope/Arterial Tourniquet (AT). PSV determines whether full blood flow cessation was achieved (i.e. 100% PSV reduction).^9^ 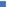 PSV APPT; 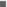 PSV Leg Rope AT.

**Figure 3:**
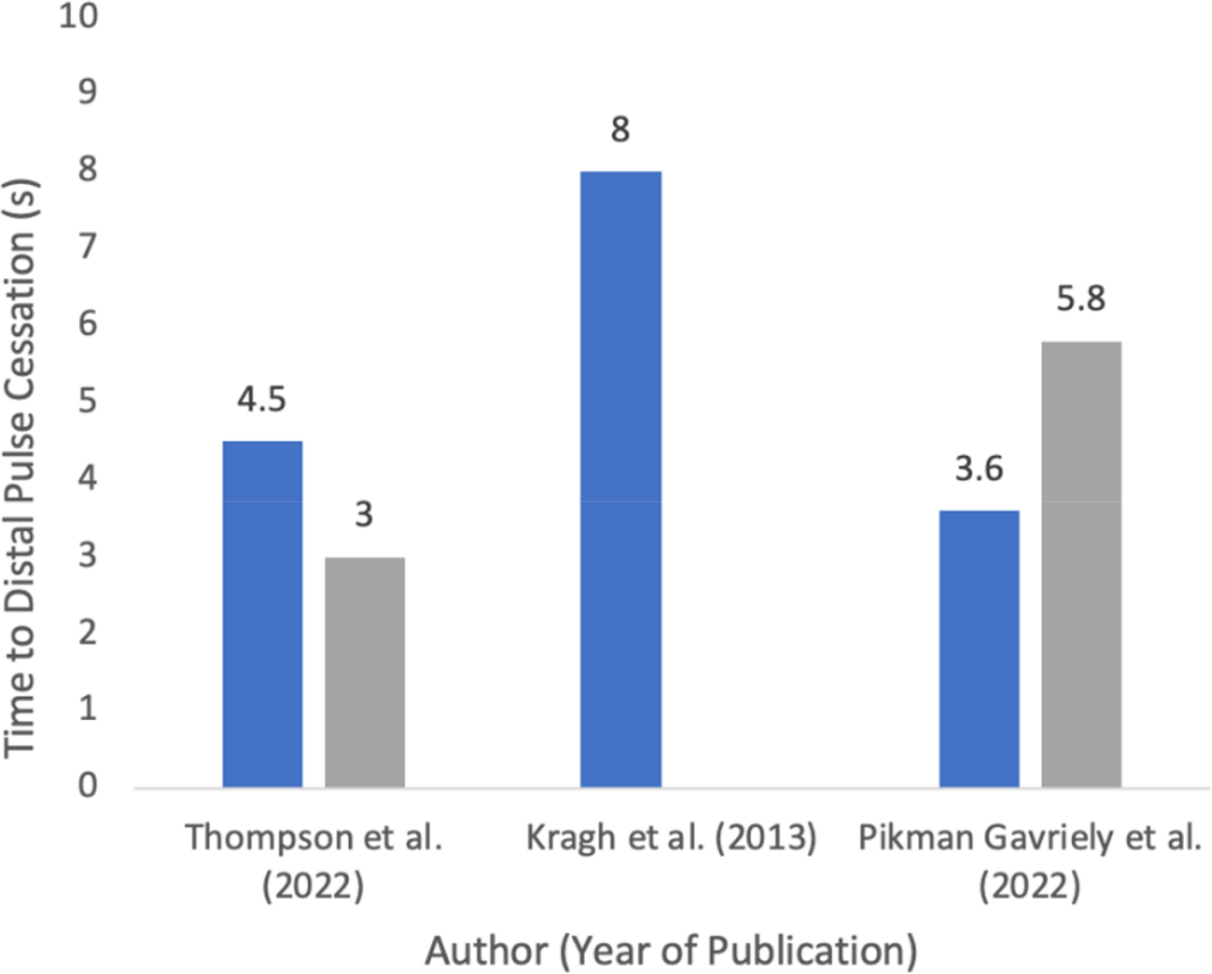
Time taken to cessation of distal pulse (100% occlusion) after APPT application. 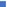 median time to distal pulse cessation (s) femoral; 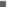 median time to distal pulse cessation (s) supraclavicular.

APPT effectiveness at different anatomical sites for blood flow cessation or reduction was assessed, and results are summarised in Table 4 as an occlusion percentage. Doppler ultrasound was employed in five studies^5^, ^7^, ^9^, ^10^, ^11^ to assess the occlusion rates. Furness *et al*.^9^ reported a complete occlusion rate of 87.5% for APPT at an average application time of 50.63 s, compared to a 50% occlusion rate for arterial tourniquets (AT) at an average time of 113.5 s (Fig. 2, Table 3). Taylor and Lamond^11^ observed significant reductions in popliteal artery peak systolic velocity (PSV) during inguinal compression compared to leg rope application (Table 3). Similarly, Slevin *et al*.^5^ found that brachial compression resulted in the highest frequency of complete vessel occlusion (73%). Pikman Gavriely *et al*.^10^ determined that the time until blood flow cessation after APPT application was 180 s, with faster cessation at the femoral pressure point than the supraclavicular. Thompson *et al*.^3^ evaluated the technique through manual radial and popliteal pulse palpation, with all participants achieving distal pulse cessation within 1 min. Swan *et al*.^7^ assessed the effectiveness of various tourniquets and found that manual digital occlusion was successful in terminating arterial pulses at different anatomical sites. Additionally, Larraga‐Garcia *et al*.^17^ and Kragh *et al*.^19^ measured the time to distal pulse cessation and achieved 100% occlusion using pressure point techniques (Fig. 3).

**TABLE 4:** Comparison table with calculated occlusion percentage (%) against study quality and limitations

Author (year)	Intervention	Vessel occluded	Occlusion benchmark	Education/information	Measurement tool	Primary outcome(s)	Key results
Pikman Gavriely *et al*. (2022)^10^	I1: Supraclavicular pressure point I2: Femoral pressure point	Supraclavicular and femoral arteries	DBFC within 120 s of pressure application	2 min oral presentation	Peak arterial flow with pulse wave Doppler US	Success rate, *n* (%)	I1:34 (97.1%) I2: 35 (100%)
						Absolute flow cessation time mean ± SD (s)	I1: 137 ± 42.6 I2: 177 ± 7.9
						Responders median perceived pain (VAS 1–10)	I1: 4 I2: 3
						Models median perceived pain (VAS 1–10)	I1: 3 I2: 2
Slevin *et al*. (2009)^5^	I1: compression at the groin I2: compression at the abdomen I3: compression at the shoulder	Femoral artery, aorta and brachial artery	Limited to 30 s application	N/A – manoeuvre completed by 2 trained research associates	MAV calculated form Doppler waveform analysis	% change of BF	I1: 78% I2: 35% I3: 97.5%
						Complete vessel occlusion	I1: 55% I2: 9% I3: 73%
Taylor and Lamond (2021)^11^	I1: Leg rope tourniquet I2: Inguinal pressure point compression.	Femoral artery	NS	Brief verbal instruction	Doppler US	Reduction in PSV	I1: 48.2% I2: 91.1%
						Complete DBFC	I1: 14 (20.5%) I2: 51 (75%)
Swan *et al*. (2009)^7^	I1: Sphygmomanometer I2: Half inch rubber tubing I3: Cloth with windlass I4: APPT arm I5: APPT cubital fossa I6: APPT groin I7: APPT knee	Brachial artery, common femoral artery and popliteal artery	Signal cessation >60 s	NS	Doppler US	Peripheral pulse arrest without pulse within 60 s	I1: *n* = 8 I2: *n* = 9 I3: *n* = 9 I4: *n* = 1 I5: *n* = 3 I6: *n* = 1 I7: *n* = 2
Avital *et al*. (2023)^6^	I1: Pressure point technique	Proximal axillary artery	NS	N/A	Vital signs	N/A	N/A

DBFC, distal blood flow cessation; I1–8, intervention # 1–8; N/A, not applicable; NS, not specified; PSV, peak systolic velocity; US, ultrasound; VAS, visual analog scale.

Charlton *et al*.^1^ conducted an SR on bleeding control methods, including APPT, haemostatic dressings, tourniquets and pressure devices, highlighting challenges in conducting high‐quality studies and performing meta‐analyses because of data heterogeneity.^1^ They suggested the adoption of arterial tourniquets as standard practice for life‐threatening bleeding yet acknowledged uncertainties in prehospital settings and for lay providers.^1^ The pilot study by Furness *et al*.^9^ suggests further evaluation of APPT's efficacy compared to arterial tourniquets through a larger, randomised crossover trial.

### Assessing the ease of application

Two studies^9^, ^17^ assessed the ease of APPT application. Furness *et al*.^9^ perceived difficulty of APPT was measured using a Likert rating scale from 0 to 10, where 0 represented no difficulty and 10 represented the most difficult (Supporting Information ‐ Appendix S3).

### Pain

Two studies^3^, ^10^ measured pain after APPT application. Thompson *et al*.^3^ measured pain scores through a numerical pain scale rating between 1 and 10, with 10 being the most severe. The median pain scores were three at the supraclavicular point and two at the femoral point. Moreover, Pikman Gavriely *et al*.^10^ had responders and models describe perceived pain level following APPT through a 10‐point visual analog scale (VAS). Median pain scores for the responders were four at the supraclavicular point and three at the femoral.^10^ Median pain scores for the models were three at the supraclavicular point and two at the femoral.^10^

### Critical appraisal

Of the nine articles included, three were considered ‘fair’ methodological quality and six were considered ‘good’ methodological quality (Table 2; Supporting Information ‐ Appendix S4).^17^ There was moderate agreement between reviewers (84%, Cohens Kappa 0.57).^20^ No articles were rated ‘poor’ methodological quality. Questions 5 and 8 were also completed poorly for a few studies,^3^, ^7^, ^17^, ^19^ which was associated with the reliability of outcome measurement and the presence of multiple pre‐ and post‐intervention outcome assessments.

### Data synthesis

The results of the transformed data are illustrated in Table 4 with a comparison to methodological quality and limitations. There is a possibility that extrapolating data in this way might have led to indirect comparison bias. Common limitations across the studies that measured occlusion were included. A meta‐analysis was not conducted because of the heterogeneity of the data and varied methodology.

## Discussion

This SR assessed APPT's efficacy in controlling major artery external haemorrhage in extremities and anatomical junctions. Although the results show APPT effectively reduces or stops distal blood flow, its superiority over other first aid techniques remains unclear because of limited studies with high levels of evidence. Most studies lack comparative techniques, show inconsistent results and have small sample sizes.

Four of the included studies^3^, ^10^, ^17^, ^19^ reported 100% blood flow cessation, yet each article had differing time benchmarks which ranged from 30 to 300 s. The longest reported time until cessation was 177 ± 7.9 s for femoral inguinal compression^10^, ^21^ Despite favourable outcomes, the variability underscores the lack of consensus in both the measurement technique and a singular time benchmark to determine success.

Furness *et al*.^9^ and Taylor and Lamond^11^ both demonstrated that APPT was more effective over arterial tourniquet or leg rope, respectively. Conversely, Swan *et al*.^7^ demonstrated relatively low success rates for APPT compared to an arterial tourniquet, with only seven out of 40 participants achieving the specific benchmark for distal pulse cessation. However, the validity of Swan *et al*.^7^ may have been compromised because of questions around whether these results were measured reliably with a potential bias to tourniquets.

Considering that emergency situations often entail prolonged waiting times for professional assistance,^22^ Kragh *et al*.^19^ emphasised a limitation to APPT as it can result in rapid fatigue in the small muscles of the digits. In this context, the research conducted by Slevin *et al*.^5^ on the modified martial arts technique, which used the knee rather than the fist, offers a potential strategy to alleviate muscular fatigue. However, there is uncertainty around the duration of APPT as well as a lack of comparison with traditional APPT using the fist or digits.^5^ To gain a more comprehensive understanding of how muscular fatigue impacts the efficacy of APPT, further trials investigating the effectiveness over extended timeframes are warranted.

Among the studies that assessed pain ratings for APPT, patient perceived pain levels post‐application was generally minimal, with scores not exceeding a four on the VAS.^3^, ^10^ Although these low pain scores are favourable, it is crucial to note that these studies^3^, ^10^ were conducted in controlled environments on healthy subjects. As such, the implications of pain arising from APPT in real‐life situations where a wound is present requires further investigation, particularly with comparison to alternative techniques.

The impact of educational approaches on APPT efficacy is evident. Furness *et al*.^9^ found that infographics improved understanding, whereas Lagarra‐Garcia *et al*.^17^ observed high haemorrhage control rates with comprehensive education (Table 3). Conversely, Swan *et al*.^7^ lacked APPT instruction details, potentially leading to reperfusion. The authors attributed reperfusion to incorrect application, highlighting the importance of education in APPT use.^7^ There is evidence to suggest a potential link between educational strategies and APPT success, as brief instruction, such as infographics or verbal guidance, could establish foundational understanding for increased efficacy. The present review demonstrates APPT's practical applicability, with studies indicating potential effectiveness through simplistic education methods for both medically trained and untrained individuals.^9^, ^17^ However, further research is needed to explore APPT's impact on individuals without medical training.

Although ANZCOR guidelines favour arterial tourniquets for haemorrhage, their evidence is weak.^23^ The pilot study by Furness *et al*.^9^ suggests further evaluation of APPT's efficacy compared to arterial tourniquets through a larger, randomised crossover trial. Although APPT could serve as a bridging technique until tourniquets are available, its preference over tourniquets remains uncertain, with potential benefits in addressing caregiver fatigue and minimising blood loss.^5^ Further research is needed to establish a gold standard for treating external haemorrhage at anatomical junctions and non‐compressible sites, considering the varying evidence and guidelines in practice.

This SR was limited by the insufficiency of the available evidence which rendered the results inconclusive. Despite the explicit search strategy, only nine articles were sourced.^3^, ^5^, ^6^, ^7^, ^9^, ^10^, ^11^, ^17^, ^19^ Moreover, the articles provided heterogeneous data with varying methodologies for outcome measurement, posing challenges in synthesising results to evaluate APPT's effectiveness in haemorrhage control. Additionally, the articles included had low levels of evidence, with most studies^3^, ^5^, ^6^, ^7^, ^10^, ^11^, ^17^, ^19^ scoring a III‐2 or IV on the NHMRC levels of evidence.^18^ Furthermore, there was a language bias of English only research incorporated, as well as the potential of publication bias whereby positive results could have been overrepresented in the literature. The limitations of this review, which have resulted from a lack of RCTs, may complicate the growing interest in APPT.

## Conclusion

The available literature indicates that APPT is effective in blood flow cessation or reduction, although there is uncertainty around its superiority over alternative first aid techniques because of the ambiguity of results which stems from low‐evidence studies and methodological heterogeneity. The impact of educational strategies, such as infographics, on APPT's success emphasises the need for standardised approaches. Further research, including additional RCTs, is necessary to evaluate APPT's efficacy as a standalone method or in combination with other techniques for immediate responders in severe bleeding situations. Despite supporting APPT's value, diverse study designs and participant characteristics necessitate standardised methodologies and larger sample sizes, including non‐medically trained individuals, for definitive conclusions.

## Supporting information


**Appendix S1:** Search strategy developed for PubMed.
**Appendix S2:** Polyglot translation searches for all additional databases used.
**Appendix S3:** Assessing the ease of application.
**Appendix S4:** The consensus results from the JBI critical appraisal checklists for the included articles.

## Data Availability

The data that supports the findings of this study are available in the supplementary material of this article.
