# Supplementary material for: Review article: Evaluating the effectiveness of arterial pressure point techniques as a first aid method for external haemorrhage control: A systematic review
Source: Emerg Med Australas. 2024 Dec 3;37(1):e14537. doi: 10.1111/1742-6723.14537 (PMC11707057; doi:10.1111/1742-6723.14537)
Supplement: Supplementary file 1 — Appendix S1: Search strategy developed for PubMed. Appendix S2: Polyglot translation searches for all additional databases used. Appendix S3: Assessing the ease of application. Appendix S4: The consensus results from the JBI critical appraisal checklists for the included articles. [file EMM-37-0-s001.docx]

**Supporting Information**

***Appendix S1: Search strategy developed for PubMed.***

("manual pressure"[Title/Abstract] OR compression[Title/Abstract] OR "pressure point"[Title/Abstract]) AND (artery[Title/Abstract] OR “blood flow” [Title/Abstract] OR bleed*[Title/Abstract] OR hemorrhage[Title/Abstract] OR haemorrhage[Title/Abstract] OR “hemorrhage” [MeSH]) AND (injuries[Title/Abstract] OR injury[Title/Abstract]) AND (limbs [Title/Abstract] OR limb [Title/Abstract] OR extremity [Title/Abstract] OR extremities [Title/Abstract] OR “extremities” [MeSH]).

***Appendix S2: Polyglot translation searches for all additional databases used.***

| **Date** | **Database** | **Search** | **Results** |
| --- | --- | --- | --- |
| 08/09/23 | CINAHL | ((TI “manual pressure” OR AB “manual pressure”) OR (TI compression OR AB compression) OR (TI “pressure point” OR AB “pressure point”)) AND ((TI artery OR AB artery) OR (TI “blood flow” OR AB “blood flow”) OR (TI bleed* OR AB bleed*) OR (TI hemorrhage OR AB hemorrhage) OR (TI haemorrhage OR AB haemorrhage) OR (MH hemorrhage+)) AND ((TI injuries OR AB injuries) OR (TI injury OR AB injury)) AND ((TI limbs OR AB limbs) OR (TI limb OR AB limb) OR (TI extremity OR AB extremity) OR (TI extremities OR AB extremities) OR (MH extremities+)) | 100 |
| 08/09/23 | Proquest   Central | ((TI,AB("manual pressure") OR TI,AB(compression) OR TI,AB("pressure point")) AND (TI,AB(artery) OR TI,AB("blood flow") OR TI,AB(bleed*) OR TI,AB(hemorrhage) OR TI,AB(haemorrhage) OR MAINSUBJECT.EXACT(hemorrhage)) AND (TI,AB(injuries) OR TI,AB(injury)) AND (TI,AB(limbs) OR TI,AB(limb) OR TI,AB(extremity) OR TI,AB(extremities)) | 94 |
| 08/09/23 | SPORTDiscuss | ((TI “manual pressure” OR AB “manual pressure”) OR (TI “compression” OR AB “compression”) OR (TI “pressure point” OR AB “pressure point”)) AND ((TI “artery” OR AB “artery”) OR (TI “blood flow” OR AB “blood flow”) OR (TI “bleed*” OR AB “bleed*”) OR (TI “hemorrhage” OR AB “hemorrhage”) OR (TI “haemorrhage” OR AB “haemorrhage”) OR DE “hemorrhage”) AND ((TI “injuries” OR AB “injuries”) OR (TI “injury” OR AB “injury”)) AND ((TI “limbs” OR AB “limbs”) OR (TI “limb” OR AB “limb”) OR (TI “extremity” OR AB “extremity”) OR (TI “extremities” OR AB “extremities”) OR DE “extremities”) | 12 |
| 08/09/23 | Embase | (‘manual pressure':ti,ab OR compression:ti,ab OR ‘pressure point':ti,ab) AND (artery:ti,ab OR’ blood flow':ti,ab OR bleed*:ti,ab OR hemorrhage:ti,ab OR haemorrhage:ti,ab OR ‘bleeding’/exp) AND (injuries:ti,ab OR injury:ti,ab) AND (limbs:ti,ab OR limb:ti,ab OR extremity:ti,ab OR extremities:ti,ab OR’ limb’/exp) | 855 |

***Appendix S3 – Assessing the ease of application***

The mean perceived difficulty for APPT application was 2.8 (SD +/- 2.8), while arterial tourniquet had a mean perceived difficulty of 3.5 (SD +/- 2.3). The median perceived difficulty for APPT application was 1.5 (IQR 4.5), while arterial tourniquet had a median perceived difficulty of 3 (IQR 3.5)^10^. Larraga-Garcia et al.^17^ used a seven-point Likert scale to assess APPT usability, with most median scores falling between six and seven (agree and strongly agree).

***Appendix S4: The consensus results from the JBI critical appraisal checklists^16^ for the included articles.***

**JBI critical appraisal for quasi experimental designs^16^.**

| **Author (Year)** | **Q1** | **Q2** | **Q3** | **Q4** | **Q5** | **Q7** | **Q8** | **Q9** | **Quality Rating** |
| --- | --- | --- | --- | --- | --- | --- | --- | --- | --- |
| **Larraga-Garcia et al. (2021)** |  |  |  |  |  |  |  |  | 50% |
| **Thompson et al. (2022)** |  |  |  |  |  |  |  |  | 50% |
| **Kragh et al. (2013)** |  |  |  |  |  |  |  |  | 50% |
| **Pikman Gavriely et al. (2023)** |  |  |  |  |  |  |  |  | 88% |
| **Slevin et al. (2019)** |  |  |  |  |  |  |  |  | 88% |
| **Taylor & Lamond (2021)** |  |  |  |  |  |  |  |  | 100% |
| **Swan et al. 2009** |  |  |  |  |  |  |  |  | 75% |

**JBI critical appraisal for RCT’s^16^.**

| **Author (Year)** | **Q1** | **Q2** | **Q3** | **Q4** | **Q5** | **Q6** | **Q7** | **Q8** | **Q9** | **Q 10** | **Q 11** | **Q 12** | **Q 13** | **Quality Rating** |
| --- | --- | --- | --- | --- | --- | --- | --- | --- | --- | --- | --- | --- | --- | --- |
| **Furness et al. (2023)** |  |  |  |  |  |  |  |  |  |  |  |  |  | 77% |

**JBI critical appraisal for case reports^16^.**

| **Author (Year)** | **Q1** | **Q2** | **Q3** | **Q4** | **Q5** | **Q6** | **Q7** | **Q8** | **Quality Rating** |
| --- | --- | --- | --- | --- | --- | --- | --- | --- | --- |
| **Avital et al. (2022)** |  |  |  |  |  |  |  |  | 88% |

**KEY:** [Green] = **Yes** [Red] = **No** [Blue] = **N/A** [Yellow] = **Unclear**

As per the methodological quality tool, ‘poor’ methodology was considered <45%, ‘fair’ methodological quality was considered as 45%-61%, and studies with ‘good’ methodological quality were >61%^16^. A Cohens Kappa analysis was completed to determine the percentage of agreement in the critical appraisal scores between the two reviewers (Z.R. & K.B.).

The mean quality rating was 75% (SD +/- 20%) with a range of 50% to 100%. The median quality rating was 88% with a 25^th^ percentile of 50% and 75^th^ percentile of 88%.
